# COVID-19 Vaccines Programs: adverse events following immunization (AEFI) among medical Clerkship Student in Jember, Indonesia

**DOI:** 10.1186/s40360-021-00528-4

**Published:** 2021-10-12

**Authors:** Supangat, Elly Nurus Sakinah, Muhammad Yuda Nugraha, Tegar Syaiful Qodar, Bagus Wahyu Mulyono, Achmad Ilham Tohari

**Affiliations:** 1 Department of Pharmacology, Faculty of Medicine, Universitas of Jember, Jl. Kalimantan No.37, Krajan Timur, Sumbersari, Kec. Sumbersari 68121 Jember, Indonesia; 2 Faculty of Medicine, University of Jember, Jember Regency, Indonesia

**Keywords:** AEFI, CoronaVac, Corona Vaccines, Covid-19, Post-vaccination, SARS-CoV-2

## Abstract

**Introduction:**

Coronavirus Disease (COVID-19) caused by Novel Coronavirus named as Severe Acute Respiratory Syndrome Coronavirus-2 (SARS-CoV-2) was declared Pandemic by The World Health Organization (WHO) and a Public Health Emergency of International Concern (PHEIC) on January 30, 2020. Many COVID-19 vaccines have been developed, including CoronaVac vaccines by Sinovac. Health care workers, along with medical clerkship students are the priority to receive the vaccine. However, the Adverse Events Following Immunization (AEFI) of the CoronaVac remains unclear. This study aims to describe and analyze the adverse events following immunization (AEFI) of COVID-19 vaccination in medical students in clerkship programs.

**Method:**

We conducted a cross-sectional study using a questionnaire to assess AEFI after CoronaVac vaccination among medical clerkship students. A Chi-Square test with 95 % of CI was used to determine whether gender correlated with symptoms of AEFI.

**Result:**

We identified 144 medical clerkship students. The most common AEFI of SARS-CoV-2 vaccinations was localized pain in the injection site during the first dose with 25 (45 %) reports and the booster dose with 34 (67 %) reports. Then followed by malaise, the first dose with 20 (36 %) reports and the booster dose with 21 (41 %) reports. Other symptoms like headache, fever, shivering, sleepiness, nausea, dysphagia, and cold were also reported.

**Conclusions:**

CoronaVac SARS-COV-2 vaccine has several mild symptoms of AEFI and not correlated with gender. Nevertheless, follow-up after vaccination is needed to prevent immunologic responses that may occur in some patients.

**Supplementary Information:**

The online version contains supplementary material available at 10.1186/s40360-021-00528-4.

## Introduction

Coronavirus Disease (COVID-19) was declared by The World Health Organization (WHO) as a pandemic caused by Novel Coronavirus (nCov) and was under Public Health Emergency of International Concern (PHEIC) on January 30, 2020 [1]. The main routes of transmission are respiratory droplets and close contact [2]. The high-risk populations that may be threatened by Sars-Cov-2 infection are cancer patients [3], sickle cell disease [4], pregnant [5], obese [6], and others. But healthcare workers are one of the most high-risk groups during this pandemic. The SARS-CoV-2 infection had been acquired by 6.9 % of healthcare workers in Torino, Italy and the others report in Belgium, Spain, and Germany was in a range between 1.6 and 9.3 % [7–10]. Meanwhile, airborne transmission routes have been reported to occur due to processes that can cause aerosol formation in patients in the hospital [11]. The clerkship program is one of the programs to train medical students in the practice of medicine in their final year of study. This program sets medical students to practice their skills directly to a patient in hospitals. During this pandemic, medical students are classified as healthcare workers with a high-risk infection of COVID-19.

As in other countries globally, Indonesia has issued a regulation about vaccination programs to combat the COVID-19 pandemic [12]. The National Immunization Technical Advisory Group (NITAG) conducted a situation assessment regarding COVID-19 vaccination and has made recommendations for access to priority groups for healthcare workers, including medical students in clerkship programs [13]. Health care workers, along with medical clerkship students are the priority to receive the vaccine. Many COVID-19 vaccines have been developed, including Pfizer, Moderna, Astrazeneca Covax In, and Indonesia health workers receive CoronaVac vaccines by Sinovac. However, the Adverse Events Following Immunization (AEFI) of the CoronaVac remains unclear. This study aims to describe and analyze the adverse events following immunization (AEFI) of COVID-19 vaccination in medical students in clerkship programs.

## Methods

### Study design and setting

We did an online cross-sectional study between February 2021. The target population was medical students in the clerkship program in Soebandi General Hospital Jember, Indonesia that underwent COVID-19 vaccination. Subjects with positive COVID-19 history, immune-compromised, pregnant, vaccine allergic history, blood clotting disorder, chronic illness (hypertension, diabetes, and other chronic diseases), autoimmune disorders, under 14 days after administration of any other vaccines, and failure to provide informed consent were excluded from this study.

Invitations to participate in the study, hosted by GoogleForms, were distributed on the WhatsApp communication platform. The survey was estimated to take∼10 min to complete. A questionnaire was constructed and developed to report the AEFI. The questionnaire uses Bahasa Indonesia. The questionnaire included sections on acceptance and post-vaccines-related symptoms or AEFI. The respondents were asked to fill the questionnaire twice (one week after the first vaccine dose and booster dose).

### Vaccine details

The vaccine details are shown in Table 1. All subjects undergo two phases of vaccination, including the first dose and booster dose within two weeks of period.

**Table 1:**
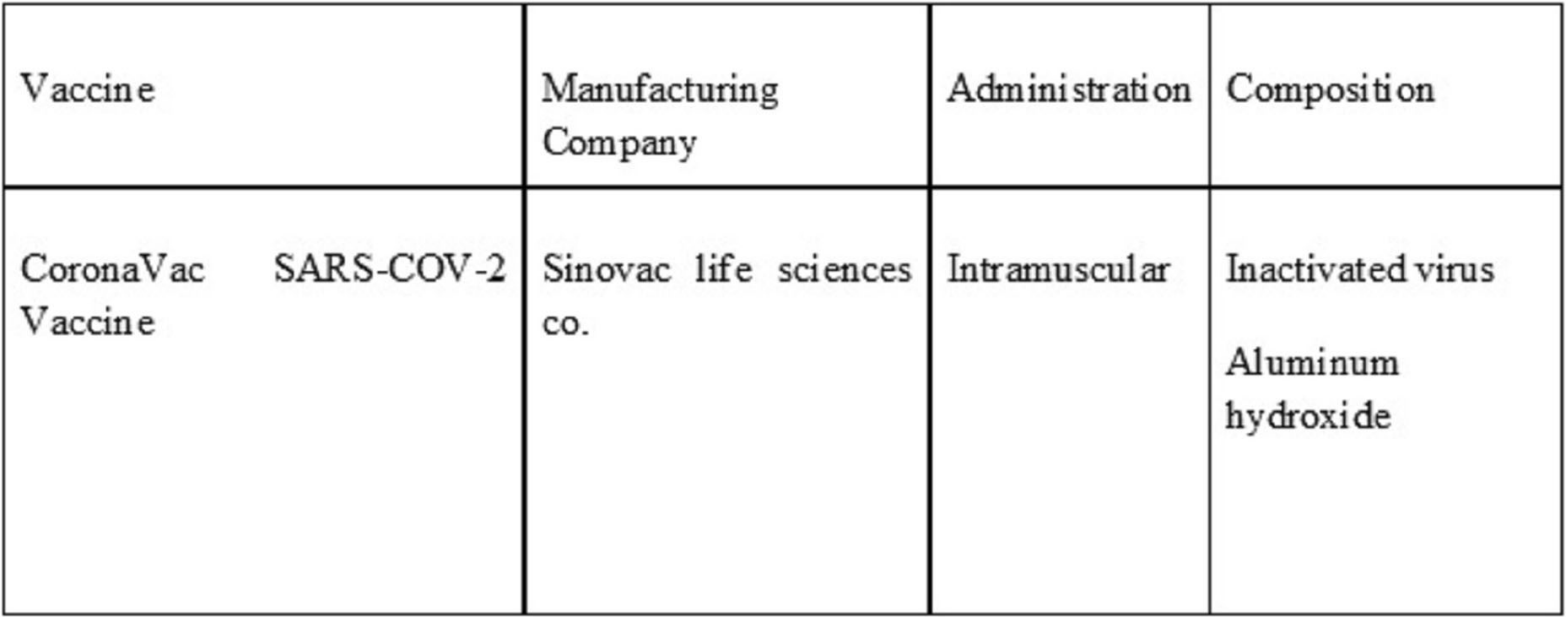
Vaccine details [14]

### Data collection and analysis

**Fig. 1:**
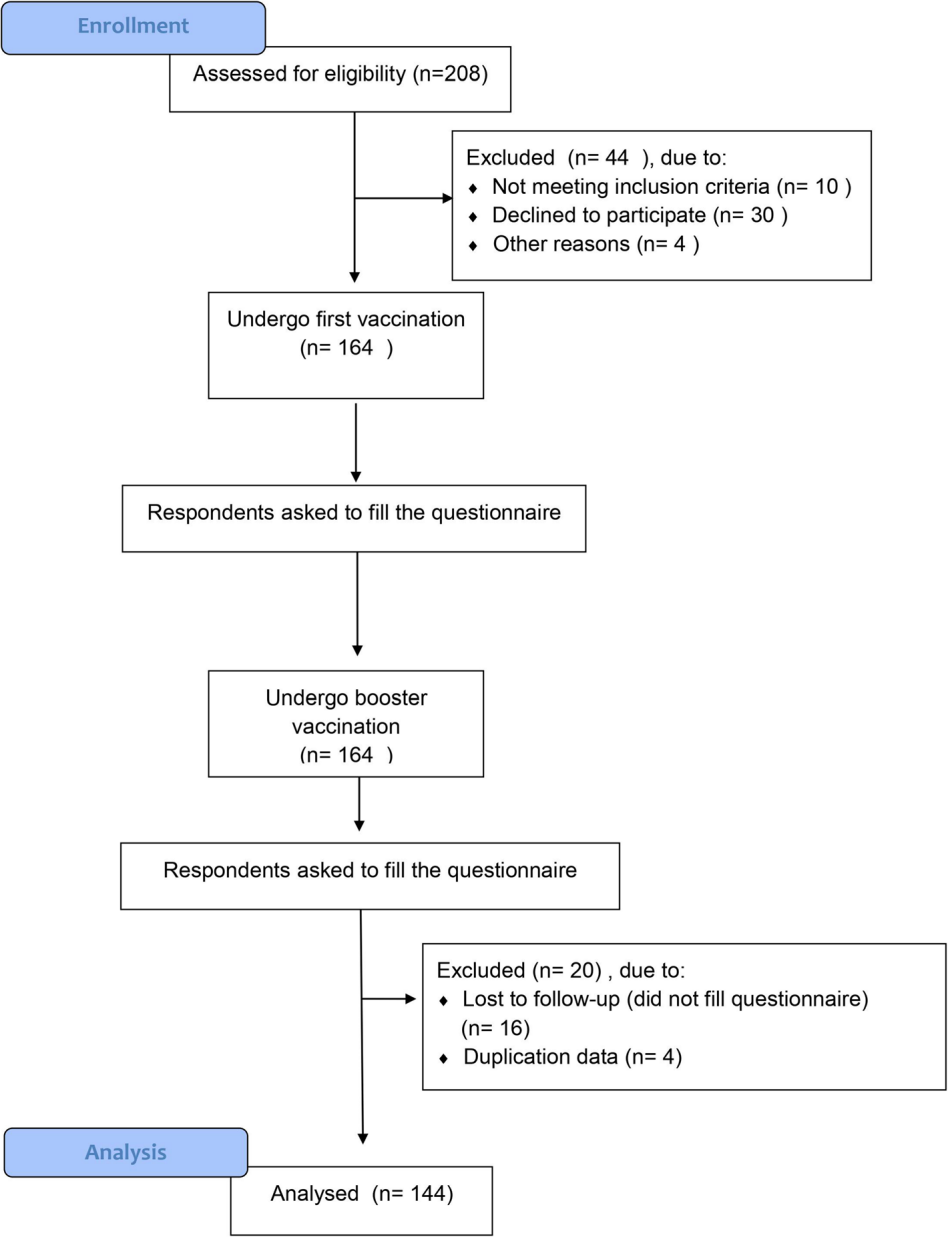
Respondents enrollment process

The questionnaire allows the respondents to enter data regarding demographic data and AEFI symptoms from the first day until one week after vaccination. We used a combined close-open question to allow the respondents to describe their AEFIs symptoms. Figure 1 shows the consort chart of the respondents selection flow of this study.

The study analysis was carried out by sorting the data into 144 cases that undergo both first dose and booster dose for vaccination. The respondent’s gender was also gathered. The questionnaire data were entered into Microsoft Excel sheets. The data analyzed statistically by using Microsoft Excel 2019 and SPSS 24. The Kolmogorov-Smirnov test was used to assess the data’s normality. Gender characteristics and events of AEFI were compared using the Chi-Square test. The statistical comparisons were performed using a predetermined significance threshold (*p* < 0.05).

### Ethics approval

 The study protocol was approved by the Institutional Review Board of the School of Medicine, Universitas Jember, Indonesia (Number 1.473/H25.1.11/KE/2021). The informed consent was conducted for each respondent before filling the questionnaire.

## Result

There were 208 medical clerkship students at Soebandi General Hospital that were assessed for COVID-19 vaccine eligibility. Amongst them, 44 (21 %) were excluded. We divided the excluded respondents into three groups. The first group, 10 clerkship students (4 %) were excluded because of not meeting the criteria of vaccination like hypertension, asthma exacerbation, diabetes, immuno-compromized and have a history of being COVID-19 positive in the past three months. The second group consists of 30 clerkship students (14 %) who declined to participate. The third group is 4 clerkship students (2 %) who were excluded due to other reasons. All included respondents were requested to fill the questionnaire to assess AEFI symptoms after vaccination.

The second follow-up began after booster vaccination. Overall, 16 out of the respondents could not be analyzed due to the loss of follow-up for the second questionnaire. We found four duplication data from all the respondents; this might happen if they fill the questionnaire twice or internet error. Total 144 medical clerkship students undergo the vaccination and this study process. In this study, the 144 homogenous respondents were 38 (26 %) men and 106 (74 %) women, with an age range between 21 and 25 years old. Homogenous respondents means that all medical students who participate as samples have the same knowledge level, they are medical clerkship students who have gone through the same educational process by completing the undergraduate stage. The respondents enrollment process is shown in Fig. 1.

**Fig. 2:**
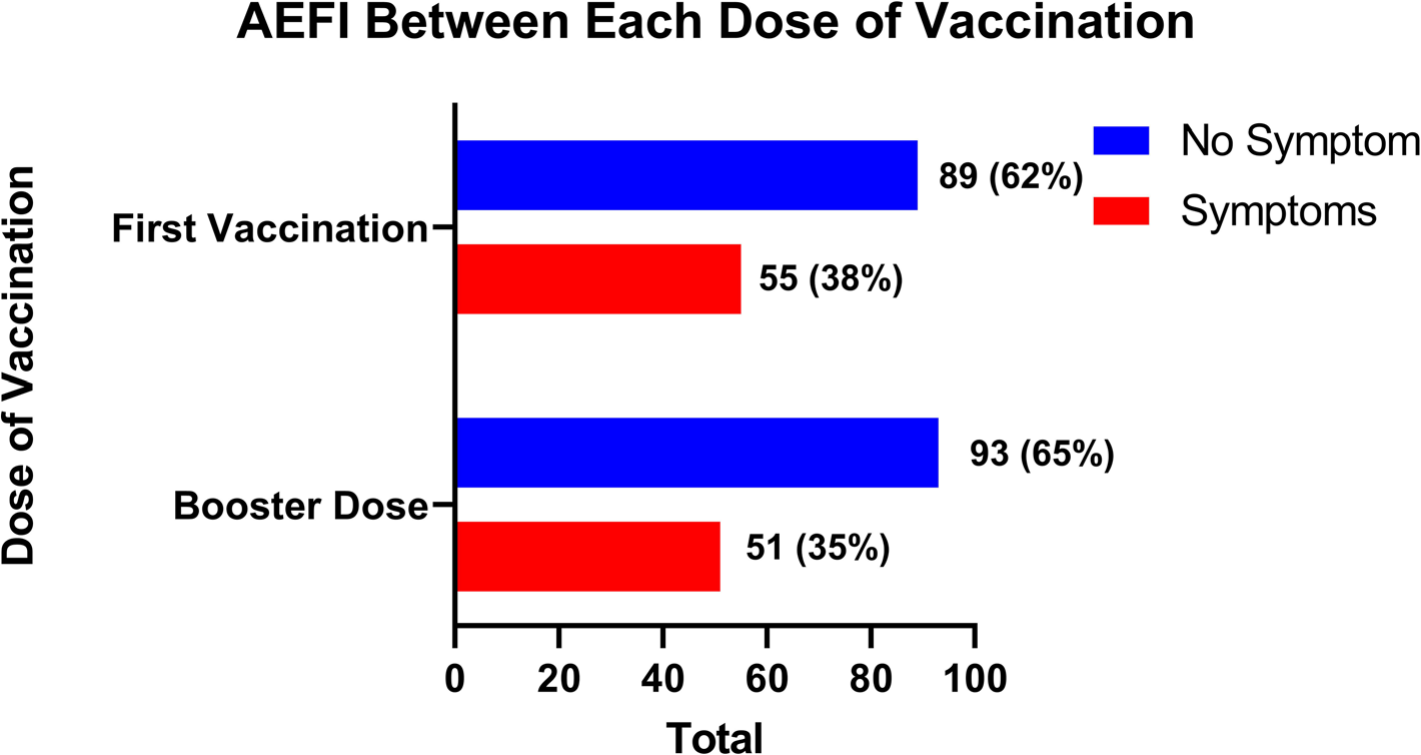
Total events of AEFI during the first and booster dose of vaccination among medical clerkship

We identified the AEFI’s report between the first and booster (Fig. 2). In the first dose, we found 55 (38 %) respondents that were reporting symptoms that indicate an AEFI. While in the booster dose, we found 51 (35 %) respondents that were reporting symptoms that indicate an AEFI.

**Fig. 3:**
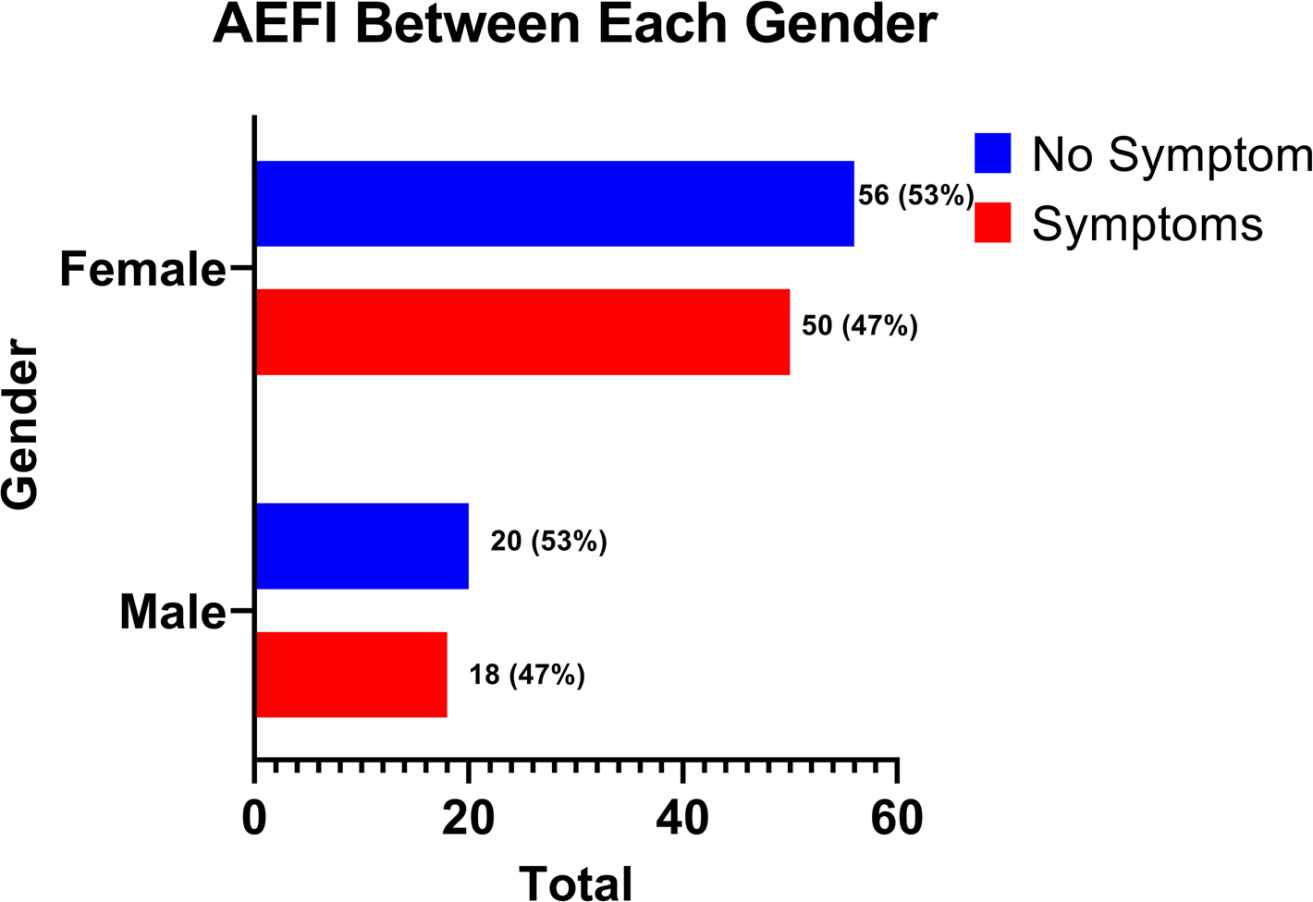
Total events of AEFI among each gender

Based on gender, we analyzed using a comparison study between the man and woman group with the correlation of AEFI using Chi-Square test. We found no difference between the man and woman groups to the AEFI (*p*-value = 0.983, IC = 95 %). The graphic between gender and the development of AEFI is shown in Fig. 3.

**Fig. 4:**
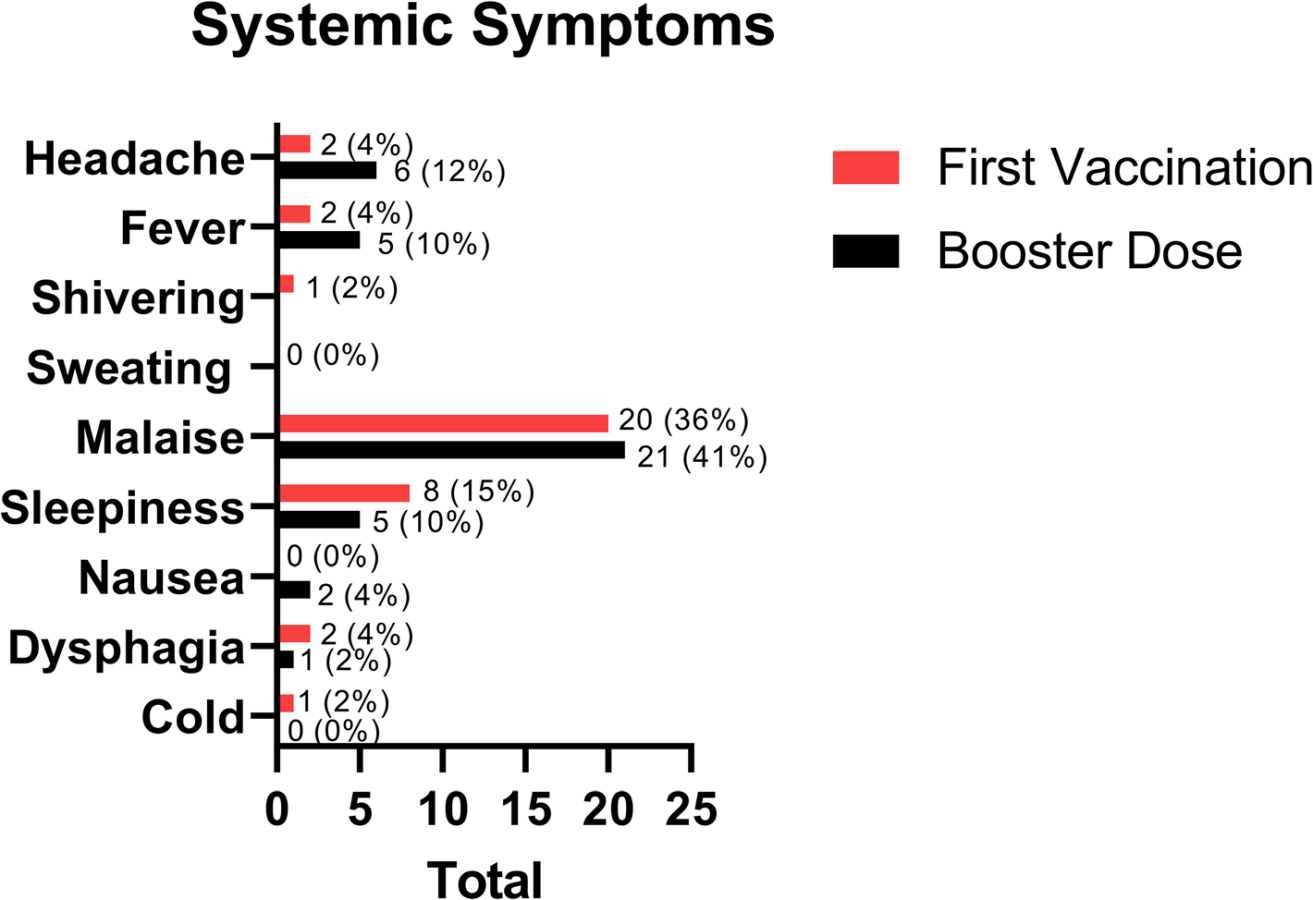
Systemic symptoms following vaccination among medical clerkship

We analyzed the typical symptoms that have been reported. There were systemic and localized effects. The systemic symptoms show a total of 36 reports in the first dose vaccination and 40 reports in the booster dose, while the localized symptoms show a total of 33 reports in the first dose vaccination and 44 reports in the booster dose. As mentioned in Fig. 4, the most common systemic AEFI symptoms of COVID-19 vaccinations was malaise with 20 (36 %) reports in the first dose and 21 (41 %) reports in the booster dose. The other types of symptoms that have been reported were: headache, fever, shivering, sleepiness, nausea, dysphagia, and cold.

**Fig. 5:**
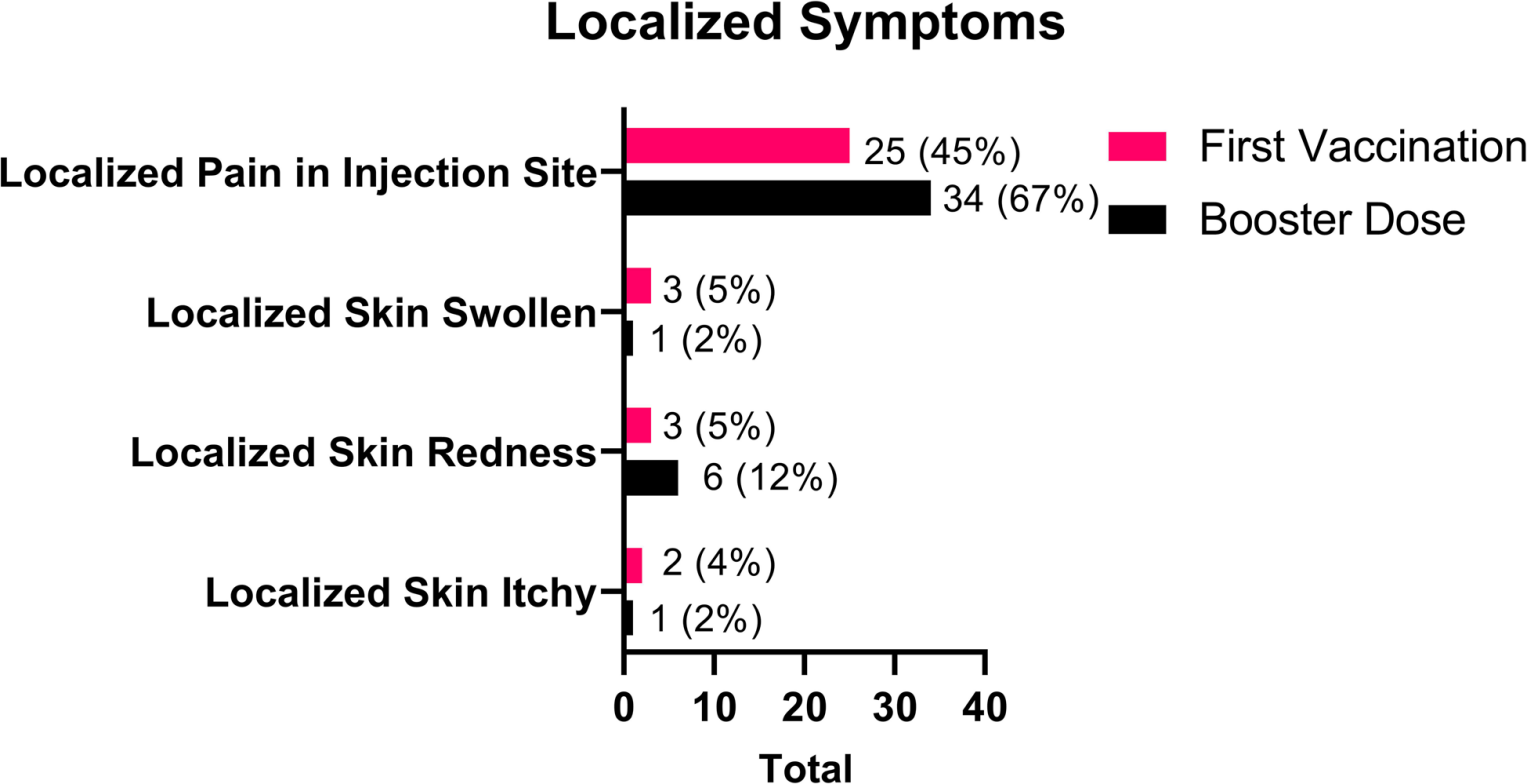
Localized symptoms following vaccination among medical clerkship

While for localized effect, as mentioned in Fig. 5, the most common localized AEFI symptoms of COVID-19 vaccinations was localized pain in the injection site during the first dose with 25 (45 %) reports and booster dose with 34 (67 %) reports. Then followed by malaise, the first dose with 20 (36 %) reports or booster dose with 21 (41 %) reports. The other types of symptoms that have been reported were localized skin swollen, the first dose with 3 (5 %) reports and booster dose with 1 (2 %) report; localized skin redness during the first dose with 3 (5 %) reports and booster dose with 6 (12 %) reports; and localized skin itchy, the first dose with 2 (4 %) reports, and the booster dose with 1 (2 %) report.

## Discussion

In this study, medical clerkship students with an average age of 21–25 years old being our subjects. Vaccination programs for the youth population would be challenged because most of them would wait until they felt the vaccine was “safe and recommended.“ Most youths in the United States sample were willing to receive a COVID-19 vaccine when they believe it is safe and recommended [14]. In our study, most of our respondents accepted vaccinations. The probable reason is information and knowledge of our subject. They are medical clerkship students that have already known about how dangerous Sars-Cov-2 infection is while they should continue their study.

In this research, we used the Corona Vac vaccine by Sinovac Biotech in China. This vaccine is inactivated SARS CoV-2 virus and uses aluminum hydroxide as an adjuvant. Three-phase I/II trials (NCT04551547, NCT04383574, NCT04352608) on 552, 442, and 744 participants, respectively, test the immunogenicity and safety of the inactivated SARS-CoV-2 vaccine. Two doses are administered 14 or 28 days apart at doses of 300 SU/0.5 mL, 600 SU/0.5 mL, and 1200 SU/0.5 mL. The measurable outcomes are the titer of neutralizing antibodies and adverse effects. The phase III trial NCT04617483 evaluates the ‘non-inferiority of the commercial scale inactivated SARS-CoV-2 vaccine’ on 1040 participants with two doses of 600 SU/0.5 mL, administered 14 days apart. The measurable outcome is the titer of neutralizing antibodies and adverse effects [15–17].

We distinguish the AEFI symptoms as systemic symptoms and localized symptoms. The symptoms reported are shown in Figs. 4 and 5. In this study, the most common AEFI of COVID-19 vaccinations was localized pain in the injection site during the first vaccination dose with 25 (45 %) reports and the booster dose with 34 (67 %) reports. The previous study conducted by Zhang shows the same common symptoms. Then followed by malaise, the first dose with 20 (36 %) reports or the booster dose with 21 (41 %) reports. The most common AEFI are several mild symptoms (grade 1) that are not described here [14]. There was no correlation between the event of AEFI symptoms with gender. Both males and females have the same chance to develop the AEFI symptoms.

We found several rare symptoms that have been reported, such as sleepiness and dysphagia. Sleepiness is the second common systemic effect in this study (15 % in the first dose and 10 % in the booster dose), it is known that all samples did not take any drugs before and after receiving the vaccine that could give a sleepiness effect. An immune response may play a role in the symptoms of dysphagia after vaccination. Nonetheless, Ishii et al. reported glossopharyngeal and vagus nerve palsies that cause dysphagia due to influenza vaccination [18]. We hypothesized the mechanism of AEFI in this vaccine might be the same. However, the pathophysiology of these symptoms remains unclear.

Our study had several limitations. First, we did not perform an immunologic test to prove the immune response from the respondents. Second, our respondents are homogenous, another AEFI might occur in different populations. Third, we just did a one-week follow-up after the vaccination. Long-term follow-up is needed to assess late symptoms of vaccination.

## Conclusions

In summary, CoronaVac SARS-COV-2 Vaccine has several mild symptoms of AEFI and not correlated with gender. Nevertheless, follow-up after vaccination is needed to prevent immunologic responses that may occur in some patients.

## Supplementary information

**Additional file 1.**

**Additional file 2.**

## Data Availability

The datasets used and/or analysed during the current study are available from the corresponding author on reasonable request.

## Abbreviations

AEFI: Adverse Events Following Immunization
