# Supplementary material for: COVID-19 Vaccines Programs: adverse events following immunization (AEFI) among medical Clerkship Student in Jember, Indonesia
Source: BMC Pharmacol Toxicol. 2021 Oct 12;22:58. doi: 10.1186/s40360-021-00528-4 (PMC8508468; doi:10.1186/s40360-021-00528-4)
Supplement: Supplementary file 1 — Additional file 1. [file 40360_2021_528_MOESM1_ESM.docx]

**COVID-19 VACCINE ADVERSE’S EFFECT QUESTIONNAIRE**

Supangat^1*^, Elly Nurus Sakinah^1^, Muhammad Yuda Nugraha^2^, Tegar Syaiful Qodar^2^, Bagus Wahyu Mulyono^2^, Achmad Ilham Tohari^2^

^1^Department of Pharmacology, Faculty of Medicine University of Jember, Indonesia

^2^Faculty of Medicine, University of Jember, Indonesia

^*^**Corresponding Author’s contact:**

Department of Pharmacology , Faculty of Medicine Universitas Jember Jl. Kalimantan No.37, Krajan Timur, Sumbersari, Kec. Sumbersari, Jember, East Java 68121 E-mail address: [drsupangat@unej.ac.id](mailto:drsupangat@unej.ac.id)

- **DEMOGRAPHIC STATE**

1. Name :
2. Age :
3. Gender :
4. Date of Vaccination :

- **SYMPTOMS**

1. Do you have any headaches after the COVID-19 vaccination?
2. Do you have a fever after the COVID-19 vaccination?
3. Do you feel tired/lethargic after the COVID-19 vaccination?
4. Do you feel chilling after the COVID-19 vaccination?
5. Do you feel sweaty after the COVID-19 vaccination?
6. Do you feel arthralgia/myalgia after the COVID-19 vaccination?
7. Do you feel malaise after the COVID-19 vaccination?
8. Do you have redness on your skin at the injection site after the COVID-19 vaccination?
9. Do you have swelling on your skin at the injection site after the COVID-10 vaccination?
10. Do you feel itchy (pruritus) on your skin at the injection site after the COVID-10 vaccination?
11. Do you have any other symptoms besides those listed above?
12. When do you feel all of these symptoms after the vaccination time?
