# Supplementary material for: COVID-19 Vaccines Programs: adverse events following immunization (AEFI) among medical Clerkship Student in Jember, Indonesia
Source: BMC Pharmacol Toxicol. 2021 Oct 12;22:58. doi: 10.1186/s40360-021-00528-4 (PMC8508468; doi:10.1186/s40360-021-00528-4)
Supplement: Supplementary file 2 — Additional file 2. [file 40360_2021_528_MOESM2_ESM.pdf]

UNEJ VERIFIED ACCOUNT

supangat Doctor <drsupangat@unej.ac.id>

Article processing charge for manuscript submitted to BMC Pharmacology and Toxicology

1 message

BioMed Central Accounts <payment@biomedcentral.com> Wed, Mar 17, 2021 at 5:18 AM
To: supangat supangat <drsupangat@unej.ac.id>

MS: 1798384264204091
COVID-19 Vaccines Programs: Adverse Events Following Immunization (AEFI) among Medical Clerkship Student in Jember, Indonesia
supangat supangat,Elly Nurus Sakinah,Muhammad Yuda Nugraha,Tegar Syaiful Qodar,Bagus Wahyu Mulyono and Achmad Ilham Tohari
BMC Pharmacology and Toxicology

Dear DR supangat

Thank you for agreeing the article processing charge for BMC Pharmacology and Toxicology. I would like to update you regarding your status with respect to the article processing charge that is normally due if a manuscript is accepted.

No payment is due on publication of this article.

The article processing charge has been waived by BioMed Central.

Kind regards,

BioMed Central Limited
The Campus
4 Crinan Street
London N1 9XW
UK
Phone: +44 (0) 20 3192 2009
Email: ORSupport@springernature.com
